# Ethanol Enhances Endothelial Rigidity by Targeting VE-Cadherin—Implications for Acute Aortic Dissection

**DOI:** 10.3390/jcm12154967

**Published:** 2023-07-28

**Authors:** Joscha Mulorz, Wiebke Ibing, Melanie Cappallo, Sönke Maximilian Braß, Kiku Takeuchi, Uwe Raaz, Isabel Nahal Schellinger, Kim Jürgen Krott, Hubert Schelzig, Hug Aubin, Alexander Oberhuber, Margitta Elvers, Markus Udo Wagenhäuser

**Affiliations:** 1 Clinic for Vascular and Endovascular Surgery, Medical Faculty and University Hospital Duesseldorf, Heinrich-Heine-University, 40225 Duesseldorf, Germany; 2 Clinic for Cardiac Surgery, Medical Faculty and University Hospital Duesseldorf, Heinrich-Heine-University, 40225 Duesseldorf, Germany; 3 CURE3D Lab, Medical Faculty and University Hospital Duesseldorf, Heinrich-Heine-University, 40225 Duesseldorf, Germany; 4 Department of Cardiology and Pneumology, University Medical Center Göttingen, Georg-August-University, 37075 Göttingen, Germany; 5 German Center for Cardiovascular Research (DZHK), Partner Site, 37075 Göttingen, Germany; 6 University Heart Center, 37075 Göttingen, Germany; 7 Clinic for Vascular and Endovascular Surgery, University Hospital Münster, 48149 Münster, Germany

**Keywords:** acute aortic dissection, endothelial tear, mechanical stability, cell adherence, risk factors

## Abstract

(1) Background: Acute aortic dissection (AAD) is caused by an endothelial entry tear followed by intimomedial delamination of the outer layers of the vessel wall. The established risk factors include hypertension and smoking. Another rising candidate risk factor is excessive alcohol consumption. This experimental study explores the effects of nicotine (Nic), angiotensin II (Ang II), and ethanol (EtOH) on human aortic endothelial cells (hAoEC). (2) Methods: HAoECs were exposed to Nic, Ang II, and EtOH at different dose levels. Cell migration was studied using the scratch assay and live-cell imaging. The metabolic viability and permeability capacity was investigated using the water-soluble tetrazolium (WST)-1 assay and an in vitro vascular permeability assay. Cell adherence was studied by utilizing the hanging drop assay. The transcriptional and protein level changes were analyzed by RT-qPCR, Western blotting and immunohistochemistry for major junctional complexing proteins. (3) Results: We observed reduced metabolic viability following Ang II and EtOH exposure vs. control. Further, cell adherence was enhanced by EtOH exposure prior to trituration and by all risk factors after trituration, which correlated with the increased gene and protein expression of VE-cadherin upon EtOH exposure. The cell migration capacity was reduced upon EtOH exposure vs. controls. (4) Conclusion: Marked functional changes were observed upon exposure to established and potential risk factors for AAD development in hAoECs. Our findings advocate for an enhanced mechanical rigidity in hAoECs in response to the three substances studied, which in turn might increase endothelial rigidity, suggesting a novel mechanism for developing an endothelial entry tear due to reduced deformability in response to increased shear and pulsatile stress.

## 1. Introduction

Acute aortic dissection (AAD) is an infrequent but once manifested critical cardiovascular disease. Patients with AAD frequently present with tearing chest pain that radiates to the back. It remains a significant health care burden with a gender-adjusted incidence of 2.53/100,000 [1] for Stanford type B aortic dissections and up to 15/1000,000 for overall AAD [2]. The health care costs of these events are substantial and often generate additional costs over subsequent years [3]. The demographic shifts occurring in all major industrial countries underline the importance for developing primary prevention concepts for cardiovascular diseases including AAD [4]. AAD entails laceration of the intima, allowing blood to flow between the layers of the media and causing delamination while typically propagating forward within the aortic wall [5].

Human aortic endothelial cells (HAoECs) form the innermost layer of blood vessels, separating blood stream components from the underlying tissue. Thus, this cell layer is of utmost interest for studying AAD at its earliest stage. An understanding of the multiprotein junctional complexes that mechanically anchor HAoECs to one another and to the basement membrane are crucial to gain mechanistic insights into the development of intimomedial tears. The endothelial barrier is mediated by tight junctions (TJs), adherens junctions (AJs), and their complexing proteins [6,7].

The most relevant risk factors for developing AAD are arterial hypertension and a history of smoking [8]. In addition, there are hints in the literature also suggesting excessive alcohol consumption as a potential risk factor [9]. The major components of AJs include cadherins, catenins, plakoglobin, and vinculin [10], whereas TJs are formed mainly by occludins and claudins [11].

As of today, little is known about how these AAD risk factors interfere with the expression levels of AJs and TJs complexing proteins and their functional properties. Therefore, this in vitro study explores how nicotine (Nic), angiotensin II (Ang II), and ethanol (EtOH) affect AJs and TJs in HAoECs and the basic functions of the cells.

## 2. Methods

### 2.1. Cell Culture

HAoECs (PromoCell, Heidelberg, Germany) were cultivated in Endothelial Cell Growth Medium (PromoCell, Heidelberg, Germany). HAoECs were cultivated at 37 °C and 5% CO_2_ (HERAcell240, Heraeus, Hanau, Germany). At 90% confluence, the cells were sub-cultured using 0.05% trypsin/0.02% ethylenediaminetetraacetic acid (EDTA) (PAN Biotech GmbH, Aidenbach, Germany). Morphological cell assessment was performed using phase-contrast microscopy (Olympus CKX41, Olympus, Shinjuku, Japan).

### 2.2. Experimental Conditions

HAoECs were exposed to 100 nM Nic (Sigma-Aldrich, St. Louis, MO, USA), 100 nM Ang II (Sigma-Aldrich, St. Louis, MO, USA), or 171 mM non-denatured EtOH (Sigma-Aldrich, St. Louis, MO, USA).

### 2.3. Cell Migration

HAoECs (1 × 10^5^) were seeded into one well of a 12-well plate and incubated in 4 mL of Endothelial Cell Growth Medium overnight. The next day, the media was removed and HAoECs were exposed to Nic, EtOH, or Ang II at the doses mentioned above for 24 h. Then, a scratch was made in the center of the plate using a 10 μL pipette tip (STARLab International GmbH, Hamburg, Germany). The plate was then placed under a live-cell imaging microscope (JuLI Br, NanoEnTek, Seoul, Republic of Korea) inside the incubator for another 24 h, taking 1 picture per hour to track the cell growth over the gap. Image analysis was performed using Fiji (Fiji is just ImageJ, NIH open source, Bethesda, MD, USA).

### 2.4. Metabolic Viability

HAoECs (1 × 10^4^ cells/well) were seeded into a 96-well plate (Bio-Rad Laboratories, Inc., Hercules, CA, USA) and 100 μL Endothelial Cell Growth Medium was added. The cells were incubated overnight at 37 °C and 5% CO_2_ inside an incubator (HERAcell240, Heraeus, Hanau, Germany). The next day, HAoECs were exposed to the respective treatments (following starvation in EC basal medium for 6 h) for 24 h. Thereafter, 10 μL of WST reagent was added to each well. After 2 h, absorbance was measured photometrically using a VICTOR™ X4 Multilabel Plate Reader (PerkinElmer, Baesweiler, Germany) at 450 nm.

### 2.5. In Vitro Vascular Permeability Assay

An in vitro vascular permeability assay (In Vitro Vascular Permeability Assay (24-Well), Millipore, Merck Chemicals GmbH, Darmstadt, Germany) was performed in a 24-well receiver plate with 24 individual hanging cell culture inserts. The inserts had a pore size of 1 μm within a transparent polyethylene terephthalate (PET) membrane. Each insert was pre-coated with rat tail collagen type I (Sigma Aldrich, St. Louis, CA, USA). The membranes allowed for apical and basolateral cell culture medium access to HAoECs. The assay was performed following the manufacturer’s instructions. In short, 1.6 × 10^5^ HAoECs were seeded and allowed to grow for 3 days to form a monolayer in close proximity to the membrane pores. The HaoECs were then exposed to the respective treatments for 24 h. Next, high-molecular-weight FITC-dextran was added for 20 min and fluorescence penetrating through the inserts was measured using a VICTOR™ X4 Multilabel Plate Reader (PerkinElmer, Baesweiler, Germany) at 450 nm/535 nm for 1.0 s.

### 2.6. Hanging Drop Assay

HaoECs were seeded onto cell culture plates and allowed to grow. When reaching 80% confluence, they were exposed to treatments for 24 h (see above). After 24 h, the HAoECs were diluted into a single cell suspension containing 4 × 10^5^ cells/mL. Then, 20 μL droplets were dropped onto the lid of a 60 mm cell culture plate filled with 3 mL of phosphate-buffered saline (PBS). The lid was carefully closed and placed in the incubator for 4 h. Then, the droplets were transferred onto a slide and three random sections of each droplet were photographed using a microscope (Axio Observer D1, Zeiss, Jena, Germany + Axiocam MRm, Zeiss, Jena, Germany). The images were analyzed using the program ZEN blue (version 3.3.89.0000, Zeiss). A subset of droplets was triturated ten times using a 10 μL pipette prior to imaging to assess cell clustering within the droplets.

### 2.7. RT-PCR and Western Blotting

To study transcriptional and protein expression changes, HAoECs were exposed to the respective treatments for 24 h. Total RNA isolation from cell cultures was performed using the RNeasy Plus Kit (Qiagen, Hilden, Germany) following the manufactures’ instructions. RNA was eluted in 50 μL RNase-free water (Qiagen, Hilden, Germany). The RNA concentration was determined spectrophotometrically using a Nanodrop (NANODROP 2000c Spectrophotometer, Thermo Scientific™, Waltham, MA, USA) at 260 and 280 nm. Complementary DNA (cDNA) synthesis was performed using high-capacity cDNA Reverse Transcription Kit (Thermo Fisher Scientific, Waltham, MA, USA) following manufacturer’s instructions, with 500 ng RNA input. The cDNA protocol consisted of the following: annealing at 25 °C for 10 min, extension at 37 °C for 120 min, and inactivation of reverse transcriptase at 85 °C for 5 min using a state-of-the-art thermocycler. For RT-PCR experiments, the cDNA input was 1 µL.

The TaqMan qRT-PCR assay was used to quantify the mRNA levels. Specific oligonucleotide primers for VE-cadherin (NM_001795), claudin-7 (NM_001307), occludin (NM_002538), nectin (NM_002856), and vimentin (NM_003380) were obtained from ORiGEN (ORiGEN, Austin, TX, USA). The data were normalized to GAPDH and fold changes were calculated using the ΔΔCt method.

For Western blotting, cells were lysed in RIPA buffer. In detail, 7.5 µg of the total protein of each sample was separated by SDS-PAGE (12% acrylamide). The proteins were transferred to 0.2 µm nitrocellulose membranes (Fisher Scientific, Waltham, MA, USA) and blocked in 1% BSA/1% nonfat dry milk for 1 h at room temperature. Next, VE-cadherin antibodies were used at a concentration of 1:1000 (Fisher Scientific, Waltham, MA, USA) and the samples were incubated at 4 °C overnight. The next day, the membranes were washed and incubated with secondary peroxidase-conjugated antibodies at a 1:20,000 dilution (Fisher Scientific, Waltham, MA, USA) for 1 h at room temperature. Chemiluminescence was detected with the Clarity Max Western ECL substrate (Bio-Rad, Hercules, CA, USA) using ChemiDoc (Bio-Rad Hercules, CA, USA) and normalized to ß-Aktin Alexa 647 (Cell signaling, Danvers, MA, USA).

### 2.8. VE-Cadherin Immunohistochemistry

A concentration of 5 × 10^4^ HAoECs per well was seeded into an 8-well slide (8-Well Chamber, removable, Sarstedt GmbH, Nümbrecht, Germany) in 400 µL EC medium and incubated overnight. Afterwards, the cells were treated with 1% EtOH (171.2 mM) in EC media for 24 h (*n* = 4) or EC media only (*n* = 4). After washing with PBS, the cells were fixed with 4% PFA and washed again with PBS. Afterwards, the cells were permeabilized using PBS containing 1% Triton X-100 (Serva Electrophoresis GmbH, Heidelberg, Germany), followed by blocking in 2% BSA for one hour at room temperature. The VE-cadherin AB (Thermo Fischer, MA, USA) was diluted (1:40) in 1% BSA and incubated overnight at 4 °C. For controls, IgG control antibody was used. After washing with PBS, the secondary AB (Alexa 594 goat anti-rabbit, Invitrogen, Thermo Fisher, MA, USA, two drops/mL PBS) was applied for one hour at room temperature in the dark. After washing with PBS, DAPI staining (1 µL/mL PBS, SIGMA-Aldrich™, MO, USA) was applied and the slides were covered with Mowiol. The slides were imaged using an Axio Observer D1 microscope (Zeiss, Jena, Germany) with an inbuilt camera (Axiocam MRm, Methods 49 Zeiss, Jena, Germany) and the program ZEN blue (version 3.3.89.0000, Zeiss) within the next 24 h. Three images of each well were taken. The exposure times were 30 ms for DAPI and 250 ms for VE-cadherin. For evaluation, cell nuclei were counted using Fiji and the intensity of VE-cadherin-associated immunofluorescence was determined. The mean grey value was normalized to the number of nuclei for each picture. The images from one well were combined and the average value was calculated.

### 2.9. Statistics

Data are presented as mean ± standard error of the mean (SEM). Individual data points are presented in the bar graphs. The differences between the experimental groups were analyzed by applying one-way ANOVA with the Bonferroni correction. A *p*-value of 0.05 was considered statistically significant.

## 3. Results

### 3.1. Cell Migration

**Figure 1:**
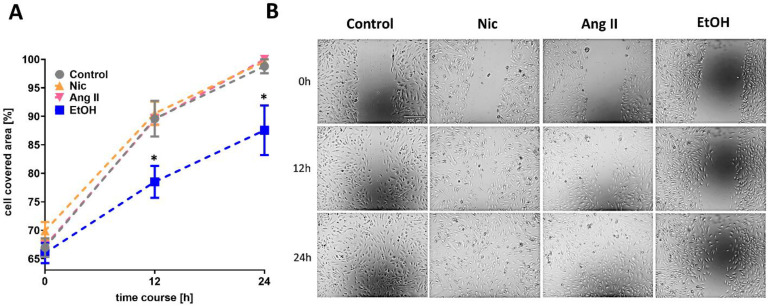
Cell migration. HAoECs were plated on 12-well-plates and exposed to nicotine (Nic) at 100 nM, angiotensin II (Ang II) at 100 nM, or ethanol (EtOH) at 171.2 mM (1% *v*/*v*). (**A**) The cell-covered area was analyzed using live-cell imaging over 24 h. EtOH caused reduced cell migration capacity vs. control. (**B**) Representative imaging of cell cultures after 12 and 24 h, showing the cell-covered area between the two cell fractions. * *p* < 0.05 vs. control applying one-way ANOVA with Bonferroni correction. (*n* = 6).

To mimic hypertension, alcohol consumption, and smoking in vitro, we exposed HAoECs to AngII, Nic, and EtOH. First, we studied whether Nic, Ang II, and EtOH exposure changed the cell migration capacity of HAoECs. When we performed a commonly used scratch assay, EtOH exposure significantly reduced cell migration, whereas Nic and Ang II exposure did not (Figure 1A). In live-cell imaging, EtOH-treated cell cultures did not show a complete closure of the gap between the two cell fractions even after 24 h, whereas there was no delayed gap closure in Nic- or Ang II-exposed cells vs. control (Figure 1B).

### 3.2. Permeability and Metabolic Activity

**Figure 2:**
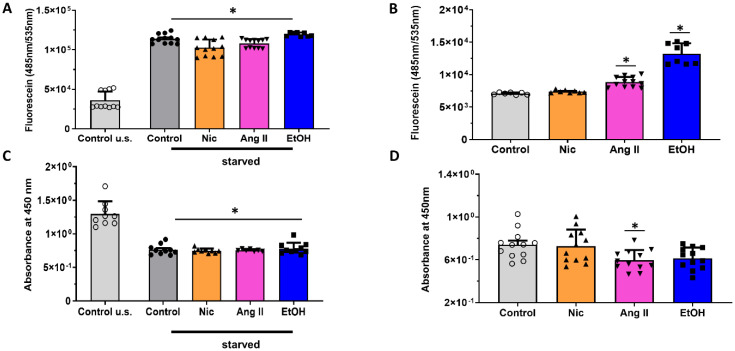
Cell permeability and metabolic viability. (**A**/**B**) Transwell assay for endothelial permeability. Human aortic endothelial cells (HAoECs) were seeded on assay inserts to form a monolayer within 3 days. Subsequently, cells were (**A**) starved for 6 h or (**B**) not starved before being exposed to the experimental conditions for 24 h. The permeability of the monolayer was tested using fluorescein isothiocyanate–dextran (FITC-dextran) by analyzing the fluorescence molecules that passed through the monolayer after 20 min. Starvation and EtOH in non-starved conditions caused an enhanced permeability. (**C**/**D**) MTT assay for metabolic viability. HAOECs were seeded in 96-well plates and (**C**) starved for 6 h or (**D**) not starved before being exposed to the experimental conditions for 24 h. Absorbance at 450 nm was measured following addition of WST-1. Starvation caused a decline in metabolic viability, whereas none of the risk factors for developing an acute aortic dissection (AAD) altered the metabolic viability in non-starved conditions. * *p* < 0.05 individual group vs. control or non-starved control (=control u.s., **A**,**C**) applying ANOVA with Bonferroni correction. (*n* = 7–12).

Next, the permeability of the endothelial cell layer and metabolic activity were evaluated. Interestingly, starvation of HAoECs resulted in increased permeability and reduced metabolic viability, underlining the significance of a stable nutritious supply for synthetic capacity and junctional integrity in these cells. Following starvation, none of the treatments further increased the permeability nor reduced the metabolic viability (Figure 2A,C). In non-starved HAoECs, the cell permeability increased upon EtOH exposure, whereas Ang II exposure reduced the metabolic cell viability vs. the control (Figure 2B,D). Of note, Nic had no effect on cell viability or permeability.

### 3.3. Mechanical Adherence

**Figure 3:**
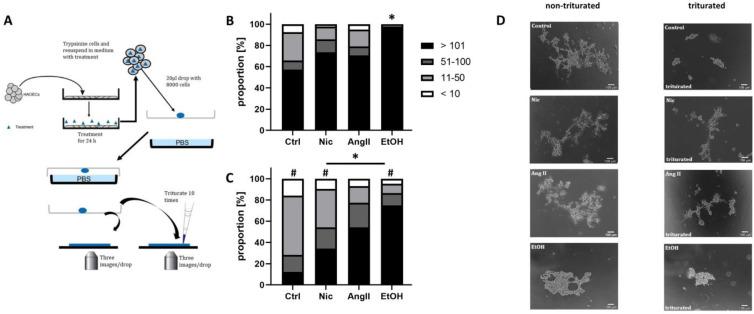
Mechanical cell adherence. (**A**) Schematic illustration of the hanging drop assay. Human aortic endothelial cells (HAoECs) were cultivated on a culture plate and then exposed to the experimental conditions for 24 h. The cells were trypsinized in cell culture medium under ongoing exposure and drops containing 8000 cells were pipetted onto the lid of a cell culture plate and incubated for 4 h. Three representative images were taken from each drop w/wo trituration to study cell adherence under increased mechanical stress. (**B**,**C**) The bar graphs show the percentage for given groups of cells in clusters. For each treatment, ±2000 cells were evaluated; data are presented as the average of the three image sections from each of six independent experiments without (**B**) and with (**C**) titration, as shown in (**D**). * *p* < 0.05 vs. control, ^#^ *p* < 0.05 vs. without titration applying one-way ANOVA with Bonferroni correction. (*n* = 6).

To analyze the mechanical adherence of HAoECs (as a marker for endothelial junctional stability), we performed a hanging drop assay (Figure 3A). We found that upon treatment with EtOH, HAoECs showed the largest cell clusters vs. the control within droplets, suggesting enhanced cell adherence (Figure 3B). Of note, enhanced intercellular cell adherence was found for all treatments following titration vs. the control. Further, reduced cell clustering vs. the non-triturated control was found in response to Nic and EtOH (Figure 3C). Interestingly, trituration itself resulted in reduced cell clustering in all treatments (Figure 3C,D).

### 3.4. Transcriptional Expression

**Figure 4:**
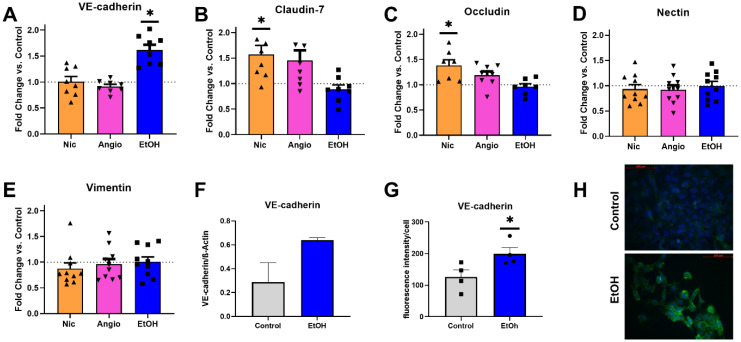
Transcriptional and protein expression levels of complexing junctional proteins. (**A**–**E**) Transcriptional levels of the complexing junctional proteins of tight and adherens junctions (TJs and AJs, respectively) following exposure to the risk factors for developing an aortic dissection (AD). The transcriptional levels were analyzed by applying the ΔΔCT method. Data are presented as fold-change vs. GAPDH. (**F**) Protein expression level of VE-cadherin following exposure to ethanol (EtOH) using Western blotting normalized to ß-Actin. (**G**) Immunohistochemistry for VE-cadherin in hAoECs normalized for fluorescence per cell, indicating marked up-regulation of VE-cadherin vs. control. (**H**) Representative images of VE-cadherin staining for quantification in G. Scale bar is 100 µm, magnification 400×. * *p* < 0.05 vs. control by applying ANOVA with Bonferroni correction. *n* = 7–10 (**A**–**E**), *n* = 3 (**F**), and *n* = 4 (**G**).

To study whether the aforementioned functional changes were associated with changes in gene expression levels of TJs and AJs, we analyzed the expression levels of the major complexing proteins in TJs, such as occluding and claudin-7, and AJs, such as VE-cadherin, nectin, and vimentin. Here, an increased transcriptional level for VE-cadherin was found upon EtOH exposure (Figure 4A). Additionally, occludin and claudin-7 expression levels were increased upon Nic exposure but not following Ang II and EtOH treatment (Figure 4B,C). We did not observe altered gene expression for nectin and/or vimentin in response to any of the treatments (Figure 4D,E). Interestingly, the described transcriptional upregulation of VE-cadherin was also found at the protein expression level, suggesting the translational persistence of regulation (Figure 4F,G).

## 4. Discussion

In our in vitro study, we found marked functional changes and altered expressional levels for major complexing proteins of TJs and AJs in HAoECs upon exposure to risk factors for the development of AAD. Our results suggest increased mechanical stability of the endothelial cell layer in response to exposure to Nic, Ang II, and EtOH. However, only EtOH treatment led to significant changes in expression levels of VE-cadherin, a major junctional complexing protein of AJs.

**Figure 5:**
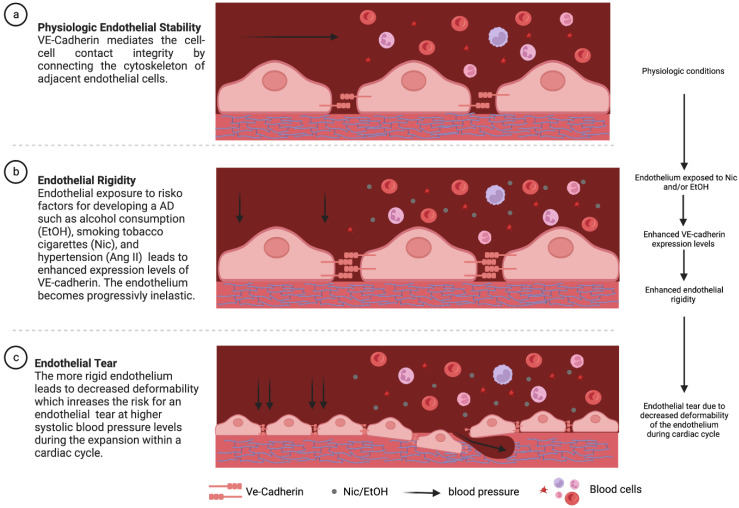
Schematic illustration of a potential pathomechanism for the development of endothelial tears in acute aortic dissection (AAD). (**a**) Under physiological conditions, adherens junctions (AJs) provide stability to the endothelial cell layer by connecting the cytoskeletons of adjacent cells. (**b**) Exposure of the endothelial cell layer to the risk factors for the development of AAD increases the expression of VE-cadherin, leading to an increase in the rigidity of the layer. (**c**) This results in the decreased deformability of the endothelial cell layer during expansion of the aorta as part of the cardiac cycle. In particular, at elevated systolic blood pressures, the endothelial cell layer “breaks” and an endothelial entry tear may be more likely to develop. Figure created with BioRender.com.

Our experimental study found marked functional changes in key cell characteristics accompanied by altered expressional levels of major complexing proteins of TJs and AJs in HAoECs upon exposure to established risk factors for the development of AAD. Further, our results comprehensively advocate for the increased mechanical stability of the endothelial cell layer upon exposure to the factors studied, which may be mediated by an increased expression of VE-cadherin, a major junctional complexing protein for AJs. Conclusively, the enhanced mechanical stability mediated via AJs and in part by TJs may enhance the rigidity of the endothelial cell layer, which in turn might increase the susceptibility for developing an endothelial tear during the expansion of the aortic diameter during the cardiac cycle (Figure 5). In other words, the question is what is more likely to break in the face of an applied expansion force, a rigid match or an eraser rubber? We here might argue for the rigid match. Although such a hypothesis remains speculative, our early experience provides preliminary evidence that such novel concept may be of importance in the mechanisms leading to the development of an endothelial entry tear.

To date, alcohol consumption is not an established risk factor for developing AAD, although more recent reports suggest a correlation [9]. In a broader context, chronic alcohol consumption has been found to elevate the blood pressure (BP) level, which is a key risk factor for developing AAD. To this regard, high-dose alcohol has a biphasic effect on BP; it decreases BP up to 12 h after consumption and increases BP >13 h after consumption [12]. Therefore, EtOH exposure may at least contribute to developing an AAD due to increased BP in the long-term. From a mechanistic point of view, EtOH seems to be an interesting candidate, given the fact that it increases permeability in endothelial cells in other locations such as lymphatic vessels or brain microvessels through, for example, Wnt/β-catenin signaling [13,14]. In line with these findings, we also observed increased permeability in HAoECs upon EtOH exposure, although we did not observe corresponding gene expression changes for the major intercellular complexing proteins of TJs such as occludin or claudin. Although this seems somewhat contradictory at first, Yu et al. found that only long-term exposure to EtOH changes the complexing protein gene expression profiles, which may at least in part explain such divergence [15]. Although it remains hypothetic, an early onset of enhanced permeability followed by a time-delayed transcriptional change may merge our findings and those from the literature, especially when also considering the reduced metabolic viability that we observed in response to EtOH exposure. Our data suggest a reduced metabolic viability by starvation itself, independent from the risk factor exposure. Since excessive alcohol consumption is often accompanied by malnutrition, this might be of relevance in the context of AAD development and may be addressed in future work.

Nevertheless, TJs only partially contribute to the mechanical stability of the endothelial cell layer; it is mainly mediated by AJs. AJs are composed of cadherin adhesion molecules interacting in a dynamic way with the cortical actin cytoskeleton [16]. To this regard, we found increased cell adherence and reduced cell migration capacity in HAoECs upon EtOH exposure, cumulatively suggesting an enhanced mechanical stability.

Various studies have reported a regulation of VE-cadherin in response to EtOH exposure in similar cell types. For instance, low concentrations of EtOH were found to up-regulate VE-cadherin expression in endothelial progenitor cells (EPCs) [17], whereas Xu et al. reported a redistribution of VE-cadherin into the intracellular compartment [18]. Of note, the effects of EtOH seem to be mediated through serval kinases such as the myosin light chain (MLC) kinase, the small GTPase Rho, and the phosphatidylinositol 3-kinase (PI3K) [19]. Although the present study did not explore mechanistic pathways, our findings further support these reports and add a similar experience in HAoECs, where we observed increased transcriptional and protein expression levels of VE-cadherin in response to EtOH. The increased rigidity, as a mechanical consequence, might increase susceptibility to the development of endothelial tears during the expansion of the aorta during the cardiac cycle due to a limited deformability. This would explain why AAD mostly occurs in the region of greatest expansion, the thoracic aortic segment.

Nic is one of the main compounds of tobacco smoke in conventional cigarettes but can also be found in E-cigarettes; the molecule has recently aroused increased scientific interest regarding its effects on the vasculature [20]. The findings of this study suggest limited functional effects for Nic exposure in HAoECs, since we did not observe significant changes in metabolic viability, cell migration capacity, or cell permeability. Nevertheless, we found Nic to facilitate cell adherence in HAoECs. Although effects of Nic on hAoECs in regard to mechanical stability are yet to be explored, the authors found enhanced stiffening of vessels upon Nic treatment in earlier murine models. Here, potential pathways such as the sirtuin-1 (SIRT1)-induced yes-associated protein (YAP) pathway were found to attenuate adverse vascular remodeling and Nic-induced vascular stiffening [21]. Moreover, elastin fragmentation via the enhanced enzymatic activity of matrix metalloproteinase (MMP) was described as a hallmark of nicotine-exposed murine aortic explants [22]. However, these stiffness-increasing effects have been described in vessel layers other than the endothelium. Our findings of increased mechanical rigidity in HAoECs mediated by enhanced cellular adherence add to these results by potentially contributing to an overall transmural enhanced stiffness and rigidity in response to Nic exposure.

In this context, previous studies have reported endothelial to mesenchymal transition (EndMT) at the vascular endothelium that was accompanied by decreased gene expression levels of VE-cadherin in thoracic murine aortic explants [23]. Additionally, Gagatet al. reported that in their study on EA.hy926 cells, Nic promoted cell movement and impaired AJs through changes in F-actin organization [24]. Taking all this into consideration, our data, along with the aforementioned studies, suggest a major effect for nicotine on the endothelium and support earlier epidemiologic data that nominate the smoking of conventional tobacco cigarettes and/or E-cigarettes as a potential candidate contributing to AAD development [25].

Hypertension is an established risk factor for developing AADs, and the role of Ang II in this process is well established [26]. Ang II can bind and activate angiotensin type 1 (AT1R) and 2 (AT2R) receptors, whereas the main pathological effects are mediated by the G protein-coupled receptor (GPCR) AT1R [27]. The primary targets of Ang II with regard to hypertension are the vascular smooth muscle cells (VSMCs). However, pleiotropic effects have also been described for Ang II in ECs by increasing intracellular reactive oxygen species (ROS) and activating nuclear factor-κB (NF-κB) [28,29]. Upon activation, proinflammatory cytokines are secreted, including tumor necrosis factor-α (TNF-α) and vascular cell adhesion molecule 1 (VCAM-1)—an important factor in endothelial cell adhesion [30]. Furthermore, Ang II activates endothelial nitric oxide synthase (eNOS), which leads to the increased availability of NO [31]. Although the mechanisms of Ang II are well described in respect to the development of hypertension, the possible effects on endothelial cell junctions have been studied to much smaller extend.

Transcriptional downregulation of VE-cadherin was described in a murine model of pulmonary hypertension [32]. In line with this, Liu et al. described the downregulation of VE-cadherin upon Ang II exposure in vascular endothelial cells [33]. When we mimicked the effects of hypertension on HAoECs by exposing these cells to Ang II, we did not observe functional alterations in key cell-specific characteristics such as cell migration capacity or metabolic viability. Additionally, we did not find altered transcriptional or translational expression levels for major complexing junctional proteins such as VE-cadherin. Interestingly, cell adherence was increased upon Ang II exposure, suggesting a functional impact of Ang II that was not reflected at a transcriptional or translational levels. The regulation of cell-adherence-mediating proteins on endothelial cells during cell recruitment in inflammatory settings is well described in the current literature, whereas linking these observations to enhanced mechanical intercellular cell adherence is a more novel finding [34,35]. Globally speaking, our findings support findings suggesting endothelial dysfunction caused by hypertension and may further nominate enhanced intercellular adherence as a more novel feature [36]. From a pathophysiological view, the relevance of hypertension in AAD development is overwhelming. Our findings suggest enhanced intercellular cell adherence as potentially being relevant in this context, although mechanisms other than targeting VE-cadherin may be of importance here. Conclusively, our results encourage further experiments that elucidate whether or not enhanced intercellular adherence contributes to the initiation of AAD by limiting endothelial deformability.

This study has some limitations. First, it has been shown that the cell morphology and functionality of endothelial cells substantially change upon laminar flow [37], so the results may be different when using non-static cell culture conditions. In addition, experiments were performed in isolated cell cultures of HAoECs in an in vitro setting, which is profoundly different from the endothelial cell environment in the human aorta. Additionally, we used constant exposure to Nic, Ang II, and EtOH, whereas HAoECs might be exposed to changing serum concentrations of these potentially hazardous substances over time in patients. Moreover, the applied concentrations of the potential risk factors for developing AD may differ substantially from human serum levels due to the study being on a monoclonal cell culture in an in vitro setting. Lastly, using Ang II to mimic hypertension and nicotine to mimic the smoking of tobacco and/or E-cigarettes might trivialize the real context, so the results might be heavily biased as we only focused on one aspect of complex biological processes.

Overall, we found marked functional changes in HAoECs upon exposure to different risk factors for developing AAD. In particular, enhanced cell adherence may provide increased mechanical stability by targeting the expressional levels of VE-cadherin, especially upon EtOH exposure. This may limit the capability of the endothelial cell layer to expand during the cardiac cycle due to enhanced rigidity, which in turn might facilitate the initiation of an endothelial entry tear in response to increased pulsatile wall stress. The present study encourages further research to prove the validity of such a hypothesis, which may also address the potential synergistic and/or antagonistic effects of the here studied risk factors in the future. In this context, different modifiable experimental parameters such as varying exposure time, different follow-up end points, and different intervals of repeated risk factor exposure could be explored to more closely mimic real-life conditions.

## Data Availability

The underlying data is available from the corresponding author upon reasonable request.

## References

[1] Melvinsdottir I.H., Lund S.H., Agnarsson B.A., Sigvaldason K., Gudbjartsson T., Geirsson A. (2016). The incidence and mortality of acute thoracic aortic dissection: Results from a whole nation study. Eur. J. Cardio-Thorac. Surg..

[2] Oberhuber A., Raddatz A., Betge S., Ploenes C., Ito W., Janosi R.A., Ott C., Langheim E., Czerny M., Puls R. (2023). Interdisciplinary German clinical practice guidelines on the management of type B aortic dissection. Gefasschirurgie.

[3] Hallberg S., Gandra S.R., Fox K.M., Mesterton J., Banefelt J., Johansson G., Levin L., Sobocki P. (2015). Healthcare costs associated with cardiovascular events in patients with hyperlipidemia or prior cardiovascular events: Estimates from Swedish population-based register data. Eur. J. Health Econ..

[4] Shetty P. (2012). Grey matter: Ageing in developing countries. Lancet.

[5] Criado F.J. (2011). Aortic Dissection: A 250-Year Perspective. Tex. Heart Inst. J..

[6] Cerutti C., Ridley A.J. (2017). Endothelial cell-cell adhesion and signaling. Exp. Cell Res..

[7] Dejana E., Orsenigo F., Molendini C., Baluk P., McDonald D.M. (2009). Organization and signaling of endothelial cell-to-cell junctions in various regions of the blood and lymphatic vascular trees. Cell Tissue Res..

[8] Dong N., Piao H., Li B., Xu J., Wei S., Liu K. (2019). Poor management of hypertension is an important precipitating factor for the development of acute aortic dissection. J. Clin. Hypertens..

[9] Goran K.P. (2009). Excessive alcohol consumption and aortic dissection: Probable but unexplored relation. Am. J. Emerg. Med..

[10] Zhuang Y., Peng H., Mastej V., Chen W. (2016). MicroRNA Regulation of Endothelial Junction Proteins and Clinical Consequence. Mediat. Inflamm..

[11] Bhat A.A., Uppada S., Achkar I.W., Hashem S., Yadav S.K., Shanmugakonar M., Al-Naemi H.A., Haris M., Uddin S. (2018). Tight Junction Proteins and Signaling Pathways in Cancer and Inflammation: A Functional Crosstalk. Front. Physiol..

[12] Tasnim S., Tang C., Musini V.M., Wright J.M. (2020). Effect of alcohol on blood pressure. Cochrane Database Syst. Rev..

[13] Laksitorini M.D., Yathindranath V., Xiong W., Parkinson F.E., Thliveris J.A., Miller D.W. (2020). Impact of Wnt/β-catenin signaling on ethanol-induced changes in brain endothelial cell permeability. J. Neurochem..

[14] Herrera M., Molina P., Souza-Smith F.M. (2021). Ethanol-Induced Lymphatic Endothelial Cell Permeability via MAP-Kinase Regulation. Am. J. Physiol. Physiol..

[15] Yu H., Wang C., Wang X., Wang H., Zhang C., You J., Wang P., Feng C., Xu G., Zhao R. (2017). Long-term exposure to ethanol downregulates tight junction proteins through the protein kinase Cα signaling pathway in human cerebral microvascular endothelial cells. Exp. Ther. Med..

[16] Cavey M., Lecuit T. (2009). Molecular Bases of Cell-Cell Junctions Stability and Dynamics. Cold Spring Harb. Perspect. Biol..

[17] Brodowski L., Schröder-Heurich B., Kipke B., Schmidt C., Von Kaisenberg C.S., Von Versen-Höynck F. (2020). Low Ethanol Concentrations Promote Endothelial Progenitor Cell Capacity and Reparative Function. Cardiovasc. Ther..

[18] Xu M., Chen G., Fu W., Liao M., Frank J.A., Bower K.A., Fang S., Zhang Z., Shi X., Luo J. (2012). Ethanol Disrupts Vascular Endothelial Barrier: Implication in Cancer Metastasis. Toxicol. Sci..

[19] Nakayama K., Hasegawa H. (2022). Blood Vessels as a Key Mediator for Ethanol Toxicity: Implication for Neuronal Damage. Life.

[20] Mulorz J., Spin J.M., Mulorz P., Wagenhäuser M.U., Deng A., Mattern K., Rhee Y.H., Toyama K., Adam M., Schelzig H. (2022). E-cigarette exposure augments murine abdominal aortic aneurysm development: Role of Chil1. Cardiovasc. Res..

[21] Hu S., Luo J., Fu M., Luo L., Cai Y., Li W., Li Y., Dong R., Yang Y., Tu L. (2021). Soluble epoxide hydrolase deletion attenuated nicotine-induced arterial stiffness via limiting the loss of SIRT1. Am. J. Physiol. Circ. Physiol..

[22] Wagenhäuser M.U., Schellinger I.N., Yoshino T., Toyama K., Kayama Y., Deng A., Guenther S.P., Petzold A., Mulorz J., Mulorz P. (2018). Chronic Nicotine Exposure Induces Murine Aortic Remodeling and Stiffness Segmentation—Implications for Abdominal Aortic Aneurysm Susceptibility. Front. Physiol..

[23] Qin W., Zhang L., Li Z., Xiao D., Zhang Y., Zhang H., Mokembo J.N., Monayo S.M., Jha N.K., Kopylov P. (2020). Endothelial to mesenchymal transition contributes to nicotine-induced atherosclerosis. Theranostics.

[24] Gagat M., Grzanka D., Izdebska M., Maczynska E., Grzanka A. (2013). Nornicotine impairs endothelial cell-cell adherens junction complexes in EA.hy926 cell line via structural reorganization of F-actin. Folia Histochem. Et Cytobiol..

[25] Kakafika A., Mikhailidis D. (2007). Smoking and Aortic Diseases. Circ. J..

[26] Howard D.P.J., Sideso E., Handa A., Rothwell P.M. (2014). Incidence, risk factors, outcome and projected future burden of acute aortic dissection. Ann. Cardiothorac. Surg..

[27] Turu G., Balla A., Hunyady L. (2019). The Role of β-Arrestin Proteins in Organization of Signaling and Regulation of the AT1 Angiotensin Receptor. Front. Endocrinol..

[28] Pueyo M.E., Gonzalez W., Nicoletti A., Savoie F., Arnal J.-F., Michel J.-B. (2000). Angiotensin II Stimulates Endothelial Vascular Cell Adhesion Molecule-1 via Nuclear Factor-κB Activation Induced by Intracellular Oxidative Stress. Arter. Thromb. Vasc. Biol..

[29] Mehta P.K., Griendling K.K. (2007). Angiotensin II cell signaling: Physiological and pathological effects in the cardiovascular system. Am. J. Physiol. Cell Physiol..

[30] Arenas I.A., Xu Y., Lopez-Jaramillo P., Davidge S.T. (2004). Angiotensin II-induced MMP-2 release from endothelial cells is mediated by TNF-α. Am. J. Physiol. Physiol..

[31] Pueyo M.E., Arnal J.-F., Rami J., Michel J.-B., de Cavanagh E.M.V., Inserra F., Dautzenberg M., Just A., Ferder M., Manucha W. (1998). Angiotensin II stimulates the production of NO and peroxynitrite in endothelial cells. Am. J. Physiol. Physiol..

[32] Nikitopoulou I., Orfanos S.E., Kotanidou A., Maltabe V., Manitsopoulos N., Karras P., Kouklis P., Armaganidis A., Maniatis N.A. (2016). Vascular endothelial-cadherin downregulation as a feature of endothelial transdifferentiation in monocrotaline-induced pulmonary hypertension. Am. J. Physiol. Cell Mol. Physiol..

[33] Liu L., Meng L., Zhang P., Lin H., Chi J., Peng F., Guo H. (2018). Angiotensin II inhibits the protein expression of ZO-1 in vascular endothelial cells by downregulating VE-cadherin. Mol. Med. Rep..

[34] Blair L.A., Haven A.K., Bauer N.N. (2016). Circulating microparticles in severe pulmonary arterial hypertension increase intercellular adhesion molecule-1 expression selectively in pulmonary artery endothelium. Respir. Res..

[35] Guedes A.F., Carvalho F.A., Moreira C., Nogueira J.B., Santos N.C. (2017). Essential arterial hypertension patients present higher cell adhesion forces, contributing to fibrinogen-dependent cardiovascular risk. Nanoscale.

[36] Drożdż D., Drożdż M., Wójcik M. (2022). Endothelial dysfunction as a factor leading to arterial hypertension. Pediatr. Nephrol..

[37] Hart D.C., van der Vlag J., Nijenhuis T. (2021). Laminar flow substantially affects the morphology and functional phenotype of glomerular endothelial cells. PLoS ONE.

